# Laparoscopic radical prostatectomy outcome data: how should surgeon’s performance be reported? A retrospective learning curve analysis of two surgeons

**DOI:** 10.3332/ecancer.2016.651

**Published:** 2016-07-06

**Authors:** Sarah Mason, Mieke Van Hemelrijck, Ashish Chandra, Christian Brown, Declan Cahill

**Affiliations:** 1 King’s College London, Division of Cancer Studies, Cancer Epidemiology Group, London, UK; 2 King’s Health Partners, London SE1 9RT, UK; 3 Royal Marsden Hospital, London SW3 6JJ, UK

**Keywords:** prostatic neoplasms, prostatectomy, learning curve, patient outcome assessment, surgeons

## Abstract

**Objective:**

To document the learning curve for the laparoscopic radical prostatectomy (LRP) procedure and discuss the optimal usage of prospectively documented outcome data for reporting a surgeon’s performance.

**Materials and methods:**

Using prospectively collected data from the first series of patients to undergo LRP by two surgeons in the same institution, linear and logistic regression multivariate analyses per 25 patients were carried out to graphically represent the surgical learning curve for operative time, blood loss, complications, length of stay (LOS), and positive margins. Surgeon A carried out 275 operations between 2003–2009; Surgeon B carried out 225 between 2008–2012.

**Results:**

Learning curves showing continuous improvement of each of the above outcomes were demonstrated for both cohorts. For surgeon A, a plateau was observed for LOS and T2 positive margins after 100 and 150 surgeries respectively. No such plateau was observed for surgeon B.

**Conclusion:**

On documenting these learning curves and discussion of the reporting methods used, we concluded that the most informative outcome measure, with the least potential observer bias was T2 positive margins. Whether as a single measure or in combination with others, this has potential for use as an objective outcome representative of improvement in a surgeon’s skill over time.

## Background

Radical prostatectomy (RP) surgical outcome data is increasingly being reported in terms of a learning curve, as influenced by surgeon-specific factors [1]. There is no definitive definition though learning curves are generally expected to plateau as a surgeon masters a technique. With the potential for altering guideline recommendations, they should be interpreted and documented with caution and appropriate statistical analysis [2].

To date, learning curves in urology have demonstrated changes in outcomes—such as operative time, blood loss, LOS, positive surgical margin (PSM) rate, complications, and cancer recurrence—to be predictive of a surgeon’s experience [3–7]. Specifically for laparoscopic RP (LRP) there is variation in the number of cases estimated to be required for competency: 51 cases according to complication rates; 110 cases according to blood loss, operative time and PSM rate [3]; and 250 according to recurrence and PSM [7, 8].

With a recent push for public reporting of a surgeon’s performance within urology [9], it is hoped that increased transparency and drive for improvement will follow. The influence of the learning curve on these reports, and the extent to which this is influenced by case mix will be of paramount consideration in documenting and interpreting a surgeon’s data with application to surgical training and clinical practice. Most surgeons do not have facilities for collecting patient reported outcomes, though this is expected to change with the demand for published outcomes increasing. The current British radical prostatectomy data set relies on a surgeon’s input data even though much of which is incomplete and difficult to verify [10, 11].

Therefore, this study involved evaluation of the optimal usage of learning curves in review of a surgeon’s performance. We aimed to use LRP outcome data from two surgeons and a discussion of the outcomes used, to determine how best to report changes in a surgeon’s performance over time. Ultimately we wanted to identify whether there are surrogate measurements available that are independent of the resource (e.g. histopathology instead of surgeon-reported data).

## Methods

### Study cohort

We used data from the first cohort of cases of extraperitoneal LRP carried out by two surgeons at King’s Health Partners. Both surgeons performed standard laparoscopic antegrade radical prostatectomies [32]. Patients data for surgeon A (275 patients) and surgeon B (225 patients) were retrieved from electronic patient records that had been recorded prospectively. Patient consent was not required as data collection was part of the clinical audit. Surgeon A operated between April 2003 and June 2009 and surgeon B between October 2008 and October 2012. The observation period and frequency of surgeries was different between the two surgeons as this reflects the uptake of radical prostatectomy in the UK with time. Both surgeons were new consultants with no independent experience so that our observations reflect a true learning curve. They were both UK trainees and completed their training with dedicated laparoscopic fellowships. Pre-operative variables included age, prostate-specific antigen (PSA), tumour stage, and Gleason score.

### Outcomes

Surgeon-dependent variables of interest were classified as operative or postoperative. Operative variables included: operative time, blood loss, and complications. Complications were reported by the operating surgeon and subsequently classified according to the 2004 Clavien-Dindo Classification [12]. Postoperative variables included: complications, LOS, and surgical margin status. The latter was assessed by three consultant histopathologists with a specialist interest in urological pathology. Prior to 2011, partial embedding of the prostate in small cassettes was the routine practice. Since 2011, the lab was progressively able to support complete embedding of the prostate in megablocks [33].

Finally, we included information on surgical stage and Gleason score. Gleason scores were divided into three categories (≤ 6, 7, and ≥ 8) as has been done in similar studies [13]. Clinical and surgical stages were classed as T0, T1a/b, T1c, T2, T3, or T4 to allow clear distinctions in analyses between patients with organ-confined and non-organ confined disease.

Apart from histopathological information, all information was prospectively collected by the surgeons.

### Statistical analysis

For each cohort of patients the follow analyses were carried out:

Patients were assigned a number according to ascending date of surgery as a representative value for a surgeon’s experience. For initial analysis of the effects of a surgeon’s experience on surgical outcomes, comparison was made between consecutive groups of 25 patients. Age, pre-operative PSA, Gleason category, and clinical stage were included in the multivariable regression model. All p-values were two-sided and considered statistically significant < 0.05. Linear regression models were used to determine the association between consecutive group numbers and mean operative time, blood loss, and LOS. These associations were graphically represented using cubic splines. Using logistic regression, we determined the association between consecutive group number and presence or absence of complications (preoperative and postoperative), positive margins, T2 positive margins, and T3/T4 positive margins, all graphically represented.

Since there was no change in practice over the study period, stage migration and date of surgery did not need to be accounted for in these multivariate models.

## Results

### Cohort details

Baseline characteristics of the patients in cohort A and B and their intra- and postoperative data are shown in Table 1. Median age was 61 (IQR 56–65) and 62 (57–66) respectively, and median presenting PSA 7 (5.6–10) and 8.3 (6.1–12.5). In both cohorts, T2 was the most common clinical stage (45% and 60% respectively) and this increased to 65% and 68% in each cohort at surgical staging. Mean operative time for surgeon A was 159 mins and for surgeon B was 225 mins; mean estimated blood loss was 268 mL and 339 mL respectively. For both surgeons mean length of a patient’s hospital stay was two days. For each outcome, there was a downward trend of continuous improvement as number of surgeries increased.

### Operative time, blood loss, and length of stay

**Figure 1.**
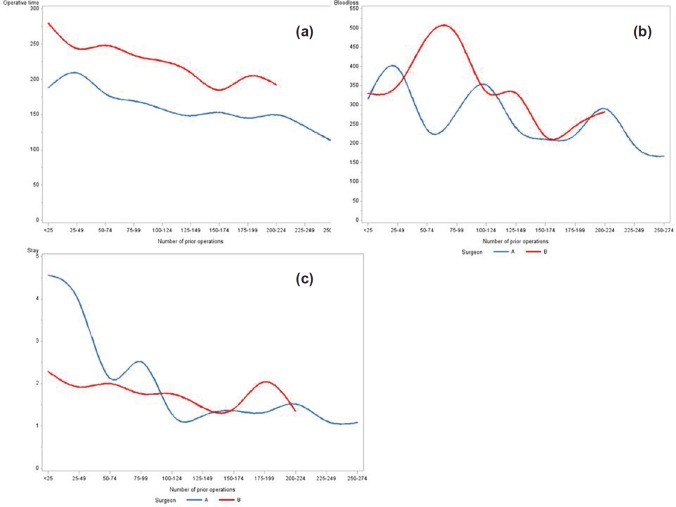
Changes by surgeon: (a) changes in operative time (min), (b) blood loss (mL) and (c) LOS (days) per 25 consecutive patients operated on by surgeon A (blue) and surgeon B (red).

Figure 1 displays the decrease in **operative time, blood loss, and LOS** observed over time. The associated crude linear regression models showed that per 25 extra surgeries, mean operative time decreased by 7.5 mins for surgeon A (p < 0.001) and 10.3 mins for surgeon B (p < 0.001). A similarly shaped trend for both surgeons is seen in Figure 1. Likewise, the crude linear regression models for blood loss demonstrated a statistically significant decrease per 25 extra surgeries of 14.1 mL (p < 0.001) for surgeon A and 19.6 mL (p < 0.001) for surgeon B. The crude linear regression model for LOS showed a decrease of 0.3 nights (p < 0.001) for surgeon A and 0.1 nights (p 0.010) for surgeon B per 25 extra surgeries. As demonstrated in Figure 1, mean LOS per 25 surgeries for surgeon A reached a plateau after 100 operations. Additional adjustments for potential confounders did not alter the above findings (results not shown).

### Surgical complications

Increasing surgeon experience was associated with a decrease in **surgical complications** (p: 0.015 and 0.095 for surgeon A and B respectively). For surgeon A, the crude logistic regression model showed that the risk of complications decreased by 19% per 25 extra surgeries (95% CI: 0.73–0.91), while for surgeon B the risk decreased by 6% per 25 extra surgeries (95% CI: 0.81–1.08). Adjustment for potentially confounding variables did not alter these findings (results not shown). On analysis of the distribution of **Clavien-Dindo** scores in each group of 25 patients, there was a statistically significant difference for the types of postoperative complications in both surgeon A and surgeon B data (p: 0.040 and 0.021 respectively), but not the types of intra-operative complications (p: 0.325 and 0.530 respectively).

### Positive margins

**Figure 2.**
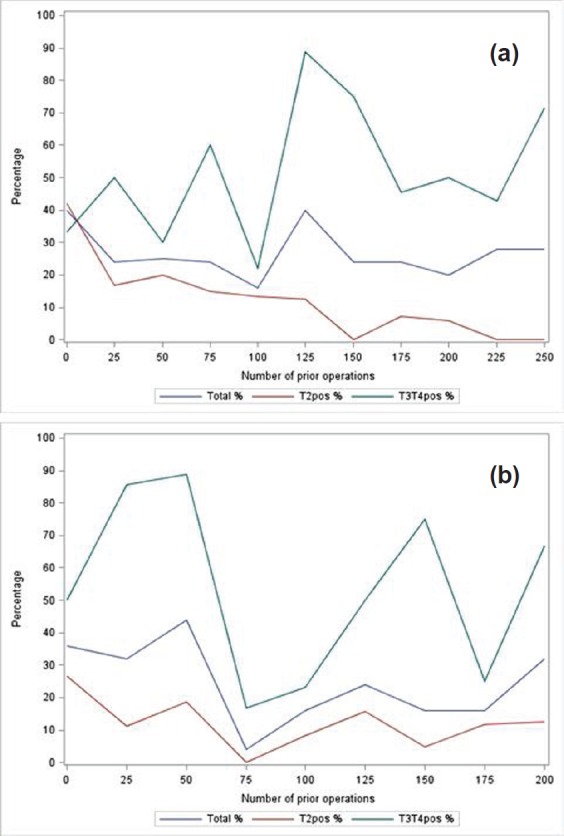
Positive margin rates: (a) positive margin rate per 25 consecutive operations (total, T2 and T3/T4 shown in blue, red, and green respectively) for surgeon A; (b) positive margin rate per 25 consecutive operations (total, T2 and T3/T4 shown in blue, red. and green respectively) for surgeon B.

Figures 2a and b report the trend for total **positive margins** as number of performed surgeries increases, stratified according to surgical stage. The logistic regression models showed a decrease in total positive margins by 2% per 25 extra surgeries for surgeon A, whereas this was 9% for surgeon B—though these associations were not statistically significant. Amongst the patients with T2 disease in surgeon A’s cohort, a statistically significant association was found between PSM rate and number of surgeries performed after adjustment for Gleason, age, PSA, and clinical stage: 29% decrease per 25 surgeries (95% CI: 0.56–0.90). A decrease of 12% (95% CI: 0.80–1.05) was reported for surgeon B. Surgeon A’s T2 positive margin rates resulted in a plateau after 150 cases (Figure 2a) whereas PSM rates were more consistent for surgeon B (Figure 2b). Such a pattern was less clear for the subgroup with T3/T4 disease.

## Discussion

Our study shows that operative time, blood loss, complications, hospital stay, and T2 margin status illustrate continuous improvement as a surgeon’s experience increases. We observed a plateau in LOS in hospital and rate of T2 PSMs.

The trends in operative time, blood loss, LOS, and complications described here parallel those which have previously been published [14, 15]. Despite some conflicting results, a recent review concluded PSM rate for open, perineal, and laparoscopic prostatectomy procedures improved with a surgeon’s skill across the learning curve [1]. Furthermore, PSM rates have been shown to be lower amongst more experienced higher volume surgeons [16, 17].

Concerning the paucity of data on how a surgical learning curve should be documented in the current context of an upcoming surgeon’s performance publication [18], it is necessary to take into account the way in which surgical outcomes are reported in an attempt to evaluate the optimal reporting method. For example, surgeon documented blood loss relies on volume estimations in fluids mixed with urine, saline, and wash, and the bleeding complications involve a method and cost-dependent balance between tissue sparing and bleeding. Furthermore, as acknowledged in the British Association of Urological Surgeons nephrectomy database, outcomes documented by operating surgeons may be subject to bias, relying on honest, complete, and consistent reporting [19]. While Hospital Episode Statistics data has advantages and can be used to verify data such as LOS, it too may be subject to certain inaccuracies when documented [20]. Within our own cohort, there was notable variation within blood loss data reported by each individual surgeon from case to case, as well as significant variation between the surgeons in reporting operative time (data not shown). Given this variation, the clinical relevance and utility of reporting changes per patient is questionable. Aside from variations in surgeon reporting, LOS also demonstrates the possible impact of other factors such as the day of the week on which a surgeon operates or the effects of other health care professionals involved in a patient’s care following surgery [15], [21].

In contrast to the above, PSM status is an outcome acquired independently of surgeon self-reporting, sensitive to continuous monitoring of a surgeon’s skill [22]. There is little evidence of significant inter-observer variability between pathologists [23]. A T2 positive margin is likely a result of iatrogenic capsular incision, being much less stage-dependent than non-organ confined cases. T3 prostate cancers are a very heterogenous group. T3 prostate cancer positive margins are more tumour dependent than surgeon dependent. The opposite is the case with T2 positive margins. Regardless of the volume of cancer within the prostate, if the gland is not incised and the cancer is organ confined, there will be a negative margin. Though there is conflicting evidence as to whether or not surgical margins status is a useful indicator of prognosis [8], [13], [24–27], we propose T2 margin rate to be most useful as described here. It is a marker of a surgeon’s performance, improving with a surgeon’s experience alongside various other surgical parameters.

One of the strengths of this study is the division of cases into groups of 25 operations—in a similar manner to the plotting of case number [28] as opposed to groups of at least 50 [1]—thereby decreasing the risk of drawing misrepresentative conclusions regarding the number of cases required for competency [29]. Multivariate analysis accounted for the positive association found in these cohorts between a surgeon’s experience and Gleason score and clinical stage. Heterogeneity between surgeons is highlighted by learning curve studies such as this, demonstrating the need for individual analysis of a surgeon’s performance with implications for both practice and training [30]. While a follow-up study might be proposed which includes the five-year biochemical recurrence (BCR) rates and continence data, it is beneficial that this study has used outcomes immediately available for a continuous record of performance. This would also be facilitated by the use of new early markers such as ultrasensitive PSA [31].

Lack of standardised measures of continence and potency impedes analysis of functional outcomes [3] and retrospectively derived data can prove unreliable. Although the surgeons in this study underwent training in a different environment to current trainees, the same outcome measures are useful and relevant for current practice. Clearly documented learning curves are likely to have future implication for the training and equipping of inexperienced surgeons in order to minimise the learning curve in surgical practice. Finally, it is a limitation that we only compared two surgeons—and future studies comparing more surgeons will provide even more insight into the use of T2 positive margin status as a marker for a surgeon’s experience.

## Conclusion

Given the need for clarity and transparency of a surgeon’s proficiency in reporting of surgeon-specific outcomes and learning curves, this study was undertaken. It shows that T2 positive margin status is the best objectively acquired parameter representative of a surgeon’s proficiency which improves with experience. All outcomes have their importance especially in terms of long-term disease-free survival and functional well being. Until outcome analysis becomes a routine part of a clinical practice. dependent on staffing and funding, it would helpful to have a surrogate to gauge performance which is least subject to reporting bias.

## Conflict of interest and funding

The authors declare that they have no conflict of interest. There are no declarations of financial support. This research received no specific grant from any funding agency in the public, commercial, or not-for-profit sectors.

## Authors’ contribution

S Mason: Literature research, data analysis, manuscript writing.

M Van Hemelrijck: Project proposal, data analysis, manuscript editing.

D Cahill: Project development, data collection, manuscript editing.

C Brown: Data collection, manuscript editing.

A Chandra: Data collection, manuscript editing.

All authors reviewed and edited the manuscript and approved the final version of the manuscript.

## Guarantor

SM

## Figures and Tables

**Table 1.** Baseline patient characteristics.

*Baseline characteristics*	Surgeon A	Surgeon B
**Median age at surgery; years (IQR)**	61 (56–65)	62 (57–66)
**Number of operations per year (%)** 2003 2004 2005 2006 2007 2008 2009 2010 2011 2012	29 (10.5) 31 (11) 30 (11) 46 (17) 61 (22) 49 (18) 29 (10.5) - - -	- - - - - 9 (4) 56 (25) 66 (29) 60 (27) 34 (15)
**Median presenting PSA; ng/mL (IQR)**	7.0 (5.6–10.0)*	8.3 (6.1–12.5)
**Clinical stage; number of patients (%)** T1a/b T1c T2 T3	11 (4) 122 (45) 123 (45) 16 (6)	8 (3.5) 71 (31.5) 135 (60.0) 11 (5.0)
**Gleason; number of patients (%)** ≤6 7 8–10	140 (52) 104 (39) 23 (9)	88 (39) 103 (46) 34 (15)
***Intra- and post-operative characteristics***		
**Mean time in min (SD)**	159 (37)**	225 (58)
**Mean blood loss in mL (SD)**	268 (212)**	339 (230)
**Intra-operative complications; number of patients (%)** Yes No **Clavien-Dindo; number (% of total intraoperative complications)** Grade I Grade II Grade III Grade IV	19 (7) 256 (93) 9 (47.0) 2 (10.5) 6 (32.0) 2 (10.5)	2 (1) 223 (99) 0 (0) 0 (0) 2 (100) 0 (0)
**Postoperative complications; number of patients (%)** Yes No **Clavien-Dindo; number (% of total postoperative complications)** Grade I Grade II Grade III Grade IV	39 (14) 236 (86) 13 (33) 13 (33) 8 (21) 5 (13)	29 (13) 196 (87) 12 (41.5) 5 (17.0) 12 (41.5) 0 (0)
**Mean stay; days (SD)**	2 (3)	2 (1)
**Surgical stage; number of patients (%)** T0 T2 T3 T4	2 (1) 175 (65) 82 (30) 11 (4)	0 (0) 153 (68) 70 (31) 2 (1)
**Mean prostate weight (SD)**	56.93 (21.72)^+^	53.77 (20.85)^++^
**Gleason score; number of patients (%)** ≤6 7 8–10	93 (36) 145 (56) 20 (8)	54 (24) 145 (64) 26 (12)
**Margins; number (%)** Total positive Total negative T2 (% of T2 cases, % of total) T3/T4 (% of T3/T4 cases, % of total)	73 (27) 201 (73) 23 (13, 8) 47 (51, 64)	55 (24) 170 (76) 18 (12, 8) 37 (51, 16)

Baseline patient characteristics and intra-/postoperative patient characteristics for total cohort of patients operated on by Surgeon A and Surgeon B. IQR: interquartile range; SD: standard deviation;

* Data missing for 4 patients;

** Data missing for 11 patients;

+ Data missing for 52 patients;

++ Data missing for 1 patient

## References

[1] Abboudi H (2014). Learning curves for urological procedures: a systematic review. BJU Int.

[2] Vickers AJ (2010). How do you tell whether a change in surgical technique leads to a change in outcome?. J Urol.

[3] Mitre AI (2013). Laparoscopic radical prostatectomy: the learning curve of a low volume surgeon. ScientificWorldJournal.

[4] Saito FJA (2011). Learning curve for radical retropubic prostatectomy. Int Brazi J Urol.

[5] Eliya F (2011). Radical perineal prostatectomy: a learning curve?. Int Urol Nephrol.

[6] Hruza M (2010). Complications in 2200 consecutive laparoscopic radical prostatectomies: standardised evaluation and analysis of learning curves. Eur Urol.

[7] Vickers AJ (2009). The surgical learning curve for laparoscopic radical prostatectomy: a retrospective cohort study. Lancet Oncol.

[8] Secin FP (2010). The learning curve for laparoscopic radical prostatectomy: an international multicenter study. J Urol.

[9] Iacobucci G (2012). Performance data on all surgeons in England will be published within two years. Brit Med J.

[10] The British Association of Urological Surgeons (2015). Surgical Outcomes.

[11] The Royal College of Surgeons England (2015). Measuring Surgical Outcomes.

[12] Dindo D, Demartines N, Clavien PA (2004). Classification of surgical complications: a new proposal with evaluation in a cohort of 6336 patients and results of a survey. Ann Surg.

[13] Vickers A (2010). The learning curve for surgical margins after open radical prostatectomy: implications for margin status as an oncological end point. J Urol.

[14] Good DW (2013). Analysis of the pentafecta learning curve for laparoscopic radical prostatectomy. World J Urol.

[15] Nelson B (2007). Comparison of length of hospital stay between radical retropubic prostatectomy and robotic assisted laparoscopic prostatectomy. J Urol.

[16] Eastham JA (2003). Variations among individual surgeons in the rate of positive surgical margins in radical prostatectomy specimens. J Urol.

[17] Vora AA (2013). Robotic-assisted prostatectomy and open radical retropubic prostatectomy for locally-advanced prostate cancer: multi-institution comparison of oncologic outcomes. Prostate Int.

[18] Khan N (2014). Measuring the surgical ‘learning curve’: methods, variables and competency. BJU Int.

[19] Henderson JM (2015). Perioperative outcomes of 6042 nephrectomies in 2012: surgeon-reported results in the UK from the British Association of Urological Surgeons (BAUS) nephrectomy database. BJU Int.

[20] Sinha S (2013). Studies using English administrative data (Hospital Episode Statistics) to assess health-care outcomes–systematic review and recommendations for reporting. Eur J Public Health.

[21] Gilmore D (2013). Timing is everything-colectomy performed on Monday decreases length of stay. Am J Surg.

[22] Touijer K (2006). Impact of a Multidisciplinary Continuous Quality Improvement Program on the Positive Surgical Margin Rate after Laparscopic Radical Prostatectomy. Eur Urol.

[23] Evans AJ (2008). Interobserver variability between expert urologic pathologists for extraprostatic extension and surgical margin status in radical prostatectomy specimens. Am J Surg Pathol.

[24] Stephenson AJ (2013). Do Margins Matter? The Influence of Positive Surgical Margins on Prostate Cancer–Specific Mortality. Urol.

[25] Tefilli MV (1998). Prognostic indicators in patients with seminal vesicle involvement following radical prostatectomy for clinically localized prostate cancer. J Urol.

[26] Barocas DA (2001). Does capsular incision at radical retropubic prostatectomy affect disease-free survival in otherwise organ-confined prostate cancer?. Urol.

[27] Klein EA (2008). Surgeon experience is strongly associated with biochemical recurrence after radical prostatectomy for all preoperative risk categories. J Urol.

[28] Thompson JE (2014). Superior Quality of Life and Improved Surgical Margins Are Achievable with Robotic Radical Prostatectomy After a Long Learning Curve: A Prospective Single-surgeon Study of 1552 Consecutive Cases. Eur Urol.

[29] Vickers AJ (2014). What are the implications of the surgical learning curve?. Eur Urol.

[30] Bianco Jr FJ (2010). Variations among experienced surgeons in cancer control after open radical prostatectomy. J Urol.

[31] Viney R (2009). Ultrasensitive prostate specific antigen assay following laparoscopic radical prostatectomy–an outcome measure for defining the learning curve. Ann R Coll Surg Engl.

[32] Bollens R (2001). Extraperitoneal laparoscopic radical prostatectomy. Results after 50 cases. Eur Urol.

[33] Association of Clinical Pathologists (2008). Guidelines for the macroscopic processing of radical prostatectomy and pelvic lymphadenectomy. J Clin Path.

